# Concomitant activation of ETS-like transcription factor-1 and Death Receptor-5 via extracellular signal-regulated kinase in withaferin A-mediated inhibition of hepatocarcinogenesis in mice

**DOI:** 10.1038/s41598-017-18190-4

**Published:** 2017-12-20

**Authors:** Panjamurthy Kuppusamy, Arumugam Nagalingam, Nethaji Muniraj, Neeraj K. Saxena, Dipali Sharma

**Affiliations:** 1 Department of Medicine, University of Maryland School of Medicine, Baltimore, MD 21201 USA; 2 Department of Oncology, Johns Hopkins University School of Medicine and the Sidney Kimmel Comprehensive Cancer Center at Johns Hopkins, Baltimore, MD 21231 USA; 3 Present Address: Early Detection Research Group, National Cancer Institute, Rockville, MD USA

## Abstract

Hepatocellular carcinoma (HCC) has the second lowest 5-year survival rate (~16%) of all tumor types partly owing to the lack of effective therapeutic agents. Withaferin A (WA) is a bioactive molecule derived from *Withania somnifera* and the present study is designed to systemically investigate the anti-HCC efficacy of WA. WA inhibited growth, migration and invasion of HCC cells. Using a phospho-kinase screening array, we discovered that WA increased phosphorylation of ERK and p38 in HCC. Further analyses revealed a key role of ERK leading to increased phosphorylation of p90-ribosomal S6 kinase (RSK) and a concomitant activation of ETS-like transcription factor-1(ELK1) and Death Receptor protein-5 (DR5) in HCC. Importantly, oral administration of WA effectively inhibited HepG2-xenografts and DEN-induced-HCC in C57BL/6 mice. Analyses of WA-treated HepG2-xenografts and DEN-induced-HCC tumors showed elevated levels of ERK, RSK, ELK1 and DR5 along with decreased expression of Ki67. *In silico* analyses of HCC, utilizing published profiling studies showed an inverse correlation between DR5 and Ki67. These data showed the efficacy of WA as an effective agent for HCC inhibition and provided first *in vitro* and *in vivo* evidence supporting the key role of a novel crosstalk between WA, ERK/RSK, ELK1, and DR5 in HCC inhibition.

## Introduction

Hepatocellular carcinoma (HCC) is the fifth most common cancer in the world and the third most common cause of cancer-related deaths across the globe^1^. The rate of incidence and mortality associated with all cancers have been declining in the USA, except for HCC which has become one of the fastest growing cause of cancer-related mortality^2^ with a 5-year survival rate of ~16%^3,4^. Local management of HCC can be successfully achieved with liver resection, ethanol injection, radiofrequency ablation, and chemoembolization when HCC is diagnosed at an early stage but despite new advancements in screening and diagnostic tools, HCC is often diagnosed at an advanced stage limiting therapeutic options. While multiple chemotherapeutic and targeted agents have been developed for other cancers, Sorafenib, an oral multikinase inhibitor, is the only approved agent for the treatment of advanced HCC. In patients with advanced HCC, sorafenib increased median survival for 3 months longer than placebo^5,6^. Clearly, it is imperative to develop novel effective therapeutic strategies for HCC to improve long-term survival of HCC patients.

Efficacy of bioactive compounds as potential cancer prevention and therapeutic agents has become apparent in recent years and many anticancer agents have been developed using natural products^7,8^.

The roots extract of medicinal plant *Withania somnifera* (commonly known as Ashwagandha or Indian winter cherry) exhibit broad spectrum pharmacological efficacy and has been successfully used in Ayurvedic medicine practice for the treatment of various ailments^9–11^. Although there are 14 bioactive compounds known as Withanolides^12,13^ in the root extract of *Withania somnifera*, Withaferin A (WA) is the most abundant and therapeutically effective withanolide with known anticancer activity^14–16^. In the present study, we specifically investigated the effect of WA on the malignant properties of hepatocellular carcinoma cells, including migration and invasion and examined the underlying molecular mechanisms. Owing to the involvement of induced-phosphorylation of key signaling proteins in cellular functions, we utilized phosphokinase arrays to investigate WA-modulated kinase pathways in HCC cells. We discovered that WA induces expression of ELK1 and DR5 via activation of ERK. Our studies show that oral administration of WA effectively inhibits HepG2-xenografts in athymic nude mice and DEN-induced HCC in C57BL/6 mice and present a novel mechanism of WA action through activation of ERK-ELK1-DR5 axis.

## Results

### Withaferin A treatment induced apoptosis and inhibited clonogenicity, anchorage-independent growth, invasion and migration of hepatocellular carcinoma cells

**Figure 1:**
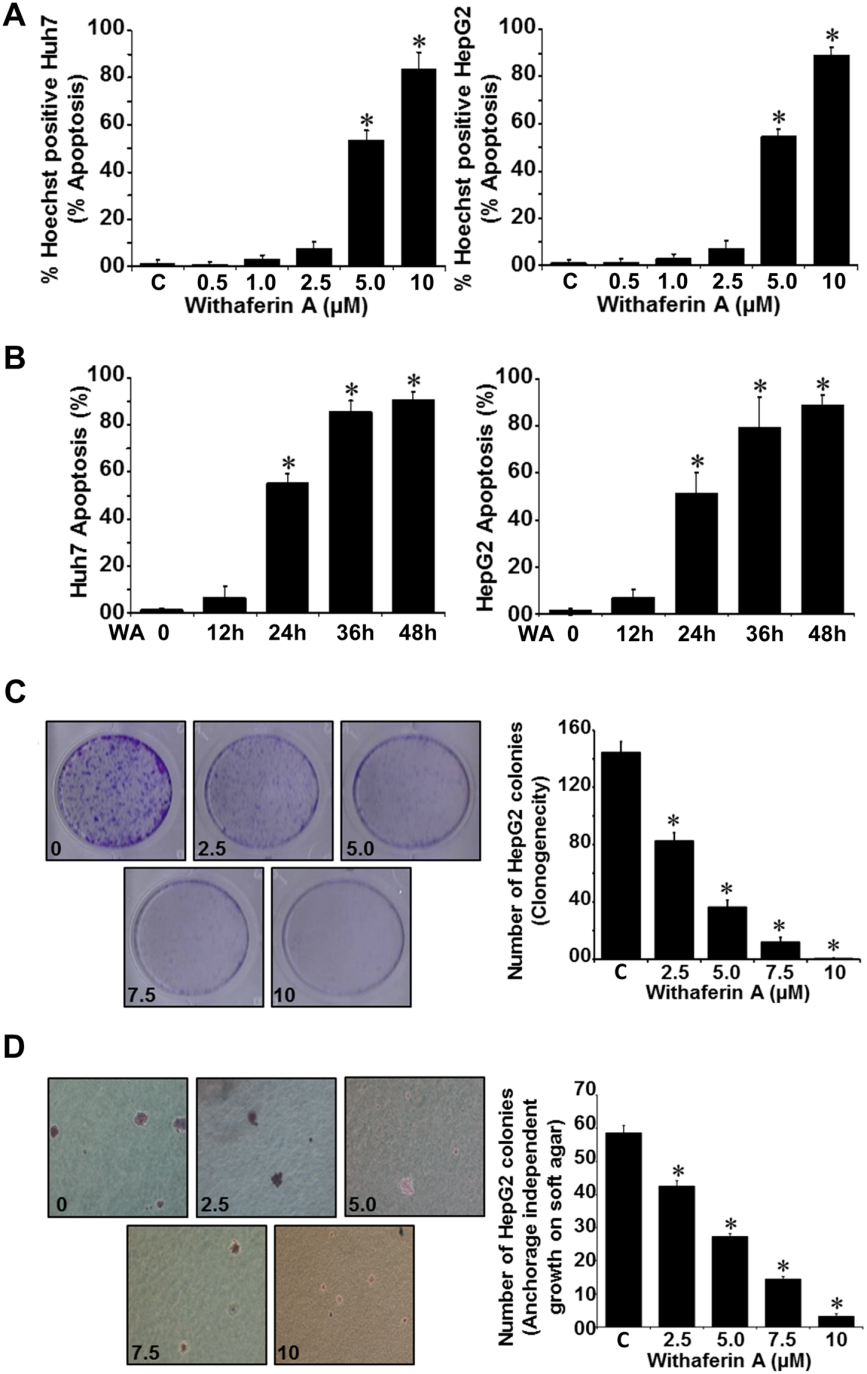
WA induces apoptosis, inhibits clonogenicity and anchorage-independent growth of HCC cells (**A**,**B**) Huh7 and HepG2 cells were treated with various concentrations of WA for 24 h as indicated (A) or treated with 5 µM WA for various time intervals as indicated (**B**) followed by Hoechst staining analyses. *p < 0.001, compared with controls (**C**). (**C**) HepG2 cells were treated with various concentrations of WA as indicated and subjected to clonogenicity assay. Histograms represent average number of colonies counted (in six micro-fields). *p < 0.005, compared with untreated cells (**C**). (**D**) HepG2 cells were subjected to soft-agar colony-formation assay in the presence of various concentrations of WA as above for 3-wks. Histograms represent average number of colonies counted (in six micro-fields). *p < 0.001, compared with untreated cells (**C**).

We examined the effect of withaferin A (WA) on HCC cells (Huh7 and HepG2). Treatment with 5 µM WA resulted in apoptotic induction in ~50% HCC cells while 10 µM WA caused apoptosis in ~80% HCC cells (Fig. 1A). Apoptotic induction was observed in ~50% HCC cells in response to 5 µM WA within 24 h and ~80–90% HCC cells showed apoptosis within 36–48 h (Fig. 1B). WA treatment resulted in dose and time dependent induction of apoptosis in Huh7 and HepG2 cells. Next, we queried whether WA inhibited the clonogenic potential and anchorage-independent growth of HCC cells. HepG2 cells showed a ~50–60% decrease in clonogenic potential in the presence of 5 µM WA while higher concentrations resulted in 80–95% inhibition (Fig. 1C). WA treatment also inhibited anchorage-independent colonies of HepG2 cells in a concentration-dependent manner with 5 µM WA resulting in ~50–60% inhibition whereas higher concentrations were more inhibitory (Fig. 1D).

**Figure 2:**
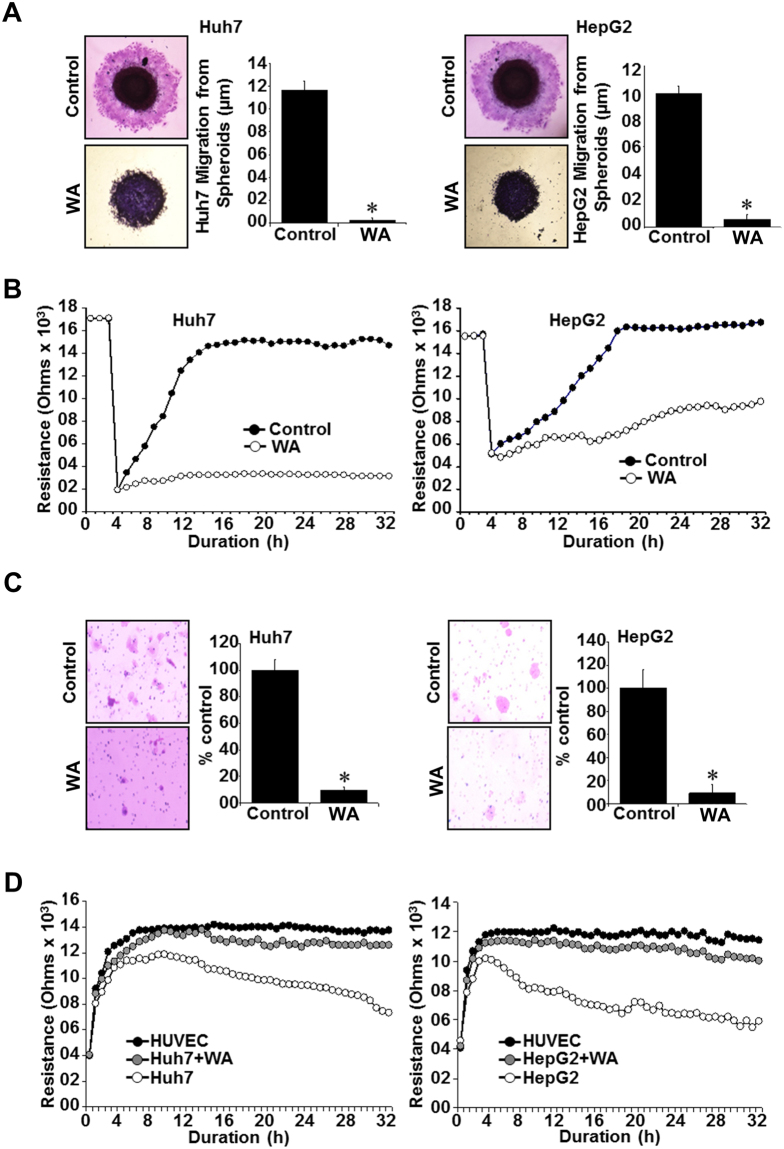
WA inhibits migration and invasion of HCC cells. (**A**) Huh7 and HepG2 cells were subjected to spheroid migration assay in the presence of 5 µM WA. HCC spheroids were photographed at 48h-post treatment. *p < 0.05. (**B**) Confluent layer of HepG2 and Huh7 cells on ECIS plates were treated with 2.5 µM WA or vehicle and subjected to an elevated voltage pulse of 40-kHz frequency at 3.5-V amplitude for 30 s. The wound was then allowed to heal from cells surrounding the small active electrode that did not undergo the elevated voltage pulse. Resistance was measured as an extent of cell migration before and after the elevated voltage pulse application and the measurements were stopped 24 h after wound healing. (**C**) Huh7 and HepG2 were subjected to matrigel-invasion assay in the presence of WA (2.5 µM) or vehicle. HCC cells that invaded the matrigel were stained (H&E) and counted. Bar graphs represent number of cells that invaded matrigel. *p < 0.05. (**D**) Resistance changes in the impedance at 4 kHz as confluent layers of HUVECs were challenged with HepG2 and Huh7 cell suspensions. The control curve of HUVEC cells received media without HepG2 or Huh7 cells. HepG2 and Huh7 cells were treated with WA (2.5 µM) or vehicle. Changes in resistance were monitored for 24 h.

Further, we questioned whether WA impacted the migratory and invasive behavior of HCC cells. To this end, Huh7 and HepG2 spheroids were treated with low-dose WA and migration of cells from the spheroids was monitored. Huh7 and HepG2 cells migrated from the spheroids in vehicle-treated conditions within 24 hours whereas WA treatment effectively inhibited migration of HCC cells (Fig. 2A). We utilized electric cell-substrate impedance sensing (ECIS) wound-healing assay for quantitative determination of the effect of WA on HCC cell migration. HCC cells treated with WA displayed decreased resistance showing that WA could inhibit migration of HCC cells on the electrode (Fig. 2B). WA treatment effectively inhibited (80–90% inhibition) invasion of HepG2 and Huh7 cells through matrigel in contrast to vehicle-treated HCC cells which showed efficient invasion through matrigel (Fig. 2C). Metastatic progression involves extravasation and intravasation of cancer cells through blood vessels. We investigated whether WA treatment could inhibit invasion of HCC cells through HUVEC cells using an ECIS-based assay. In this assay, HUVEC cell layer was established on ECIS chamber and challenged with HCC cells. A decrease in resistance was observed in chambers with untreated Huh7 and HepG2 cells showing direct interactions of the HCC cells with HUVEC cells and extravasation of HCC cells to the substratum. Interestingly, Huh7 and HepG2 cells could not invade through HUVEC cell layer in the presence of WA treatment as indicated with high resistance levels that remained largely unaltered similar to HUVEC cell layer controls (Fig. 2D).

### Distinct withaferin A mediated alterations in phosphorylation of signaling mediators were observed in phosphokinase array

**Figure 3:**
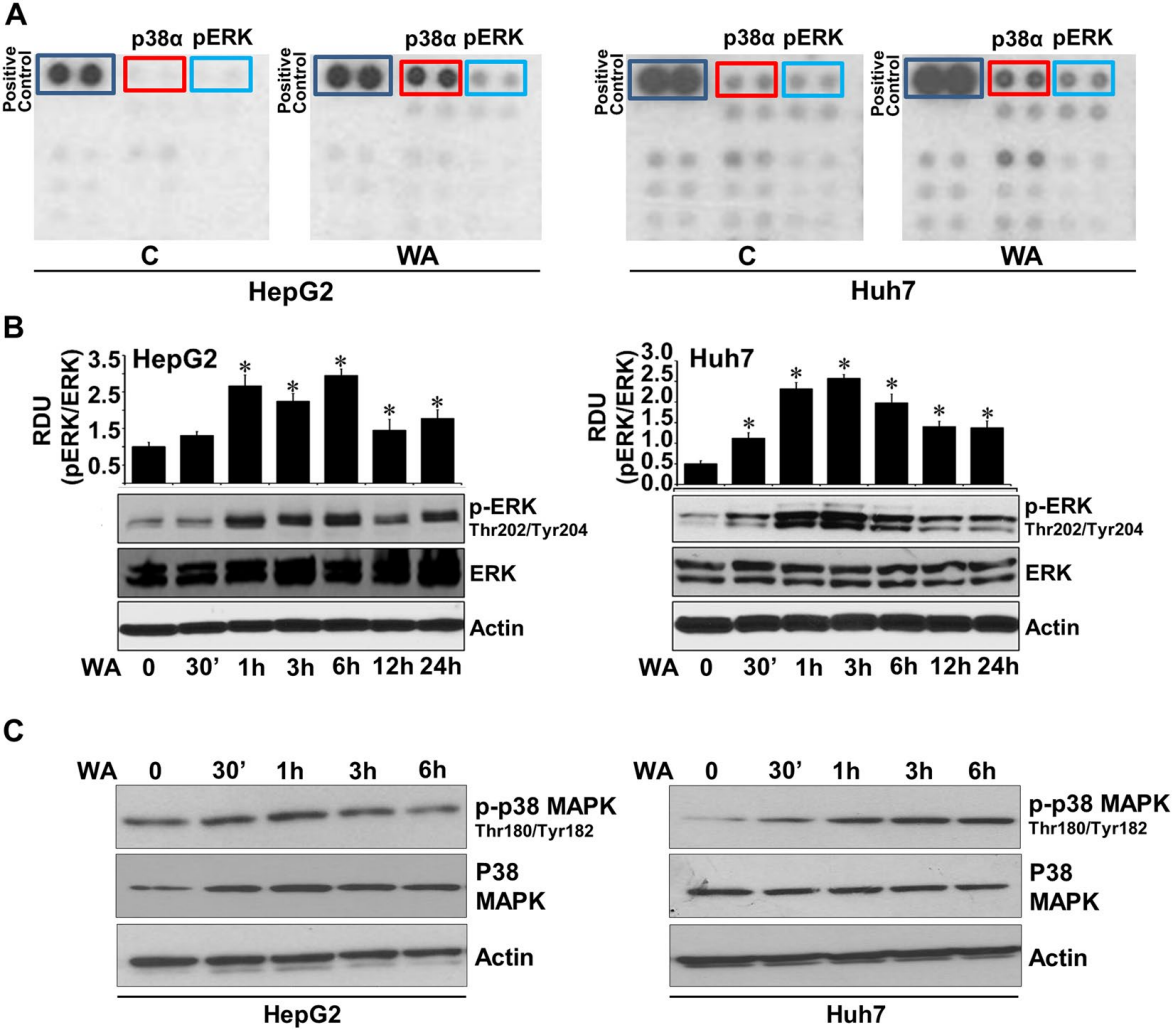
Human phosphokinase array reveals alteration in phosphorylation of kinases upon WA treatment. (**A**) MCF7 and MDA-MB-231 breast cancer cells were treated with 5 µM WA and subjected to Human phospho-antibody array analyses. Relative levels of protein phosphorylation (normalized intensity for each antibody) were calculated for each untreated and treated sample. (**B**) Immunoblot analysis of phosphorylated-ERK (pERK) and total ERK in Huh7 and HepG2 cells treated with 5 µM WA as indicated. Bar graphs show relative density units of pERK/ERK. *p < 0.005, compared with 0 hour control cells. (**C**) Total lysates of Huh7 and HepG2 cells treated with 5 µM WA were immunoblotted with phosphorylated-p38 and total p38 as indicated. Actin was used as a loading control.

Biological effects of extracellular stimuli can be mediated via phosphorylation/dephosphorylation events regulating activation/inhibition of specific kinases and their downstream signaling cascades, which can consequently control various cellular functions. To ascertain signaling networks involved in WA function in HCC, we utilized a phosphoprotein-array encompassing 46 specific Ser/Thr/Tyr phosphorylation sites of 38 selected proteins. Huh7 and HepG2 cells were treated with 5 µM WA for 6 h and subjected to phospho-protein analysis. We discovered that WA treatment increased phosphorylation of p38 mitogen-activated protein kinase (p38α MAPK) and extracellular signal-regulated kinase (ERK) in Huh7 and HepG2 cells (Fig. 3A). Phosphoprotein array results were corroborated in independent immunoblot analyses. Immunoblot analyses confirmed a temporal increase in phosphorylation of p38α in HCC cells upon WA treatment (Fig. 3C). Activation of p38α MAPK pathway has been linked to caspase-activation and induction of apoptotic cascade in HCC and p38 inhibition decreases caspase activity and cell death in response to docetaxel-based chemotherapy^17,18^. Huh7 and HepG2 cells also showed increased ERK phosphorylation levels within 1-hour post-WA treatment whereas total ERK levels remained unaltered corroborating phosphokinase array results (Fig. 3B). This was an intriguing finding as WA exhibited anti-HCC potential in functional assays but induced phosphorylation of ERK, a major survival signaling pathway, hence we further explored the role and importance of ERK in WA-mediated inhibition of HCC.

**Figure 4:**
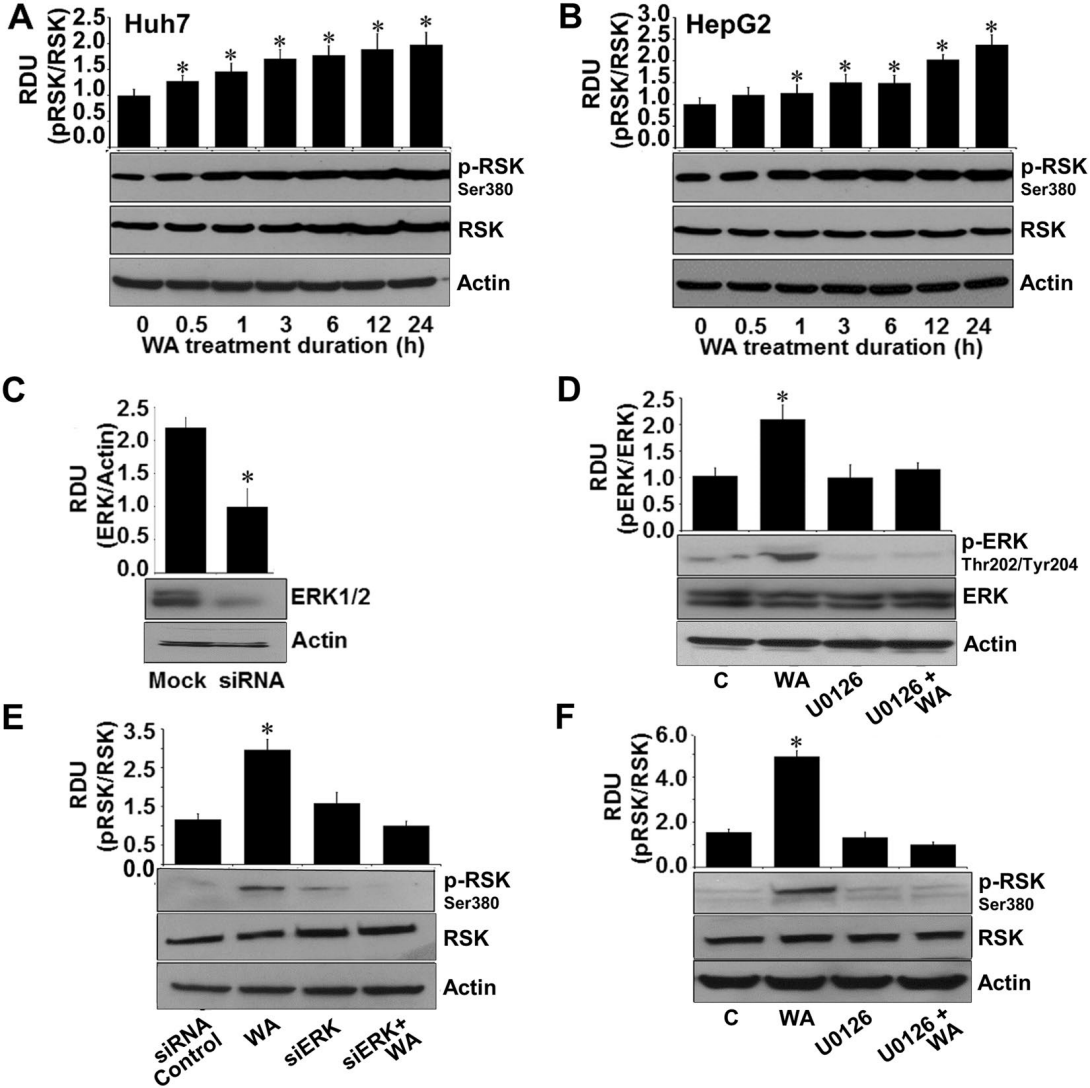
WA activates RSK in an ERK-dependent manner. (**A**,**B**) Immunoblot analysis of phosphorylated-RSK-Ser380 (pRSK) and total RSK in Huh7 and HepG2 cells treated with 5 µM WA as indicated. *p < 0.001, compared with 0 hour control cells. (**C**) HCC cells were transiently transfected with siERK-siRNAs for 48 h followed by immunoblot analysis of ERK levels. *p < 0.001, compared with mock control cells. (**D**) HCC cells were pretreated with 10 µM U0126 for 2 hours followed by treatment with 5 µM WA. Total lysates were immunoblotted for pERK and total ERK expression. *p < 0.005, compared with control cells (**C**). (**E**) HCC cells were transiently transfected with siERK-siRNA and treated with 5 µM WA as indicated and followed by immunoblot analysis of pRSK and total RSK expression. *p < 0.001, compared with siRNA control cells. (**F**) HCC cells were pretreated with 10 µM U0126 for 2 hours followed by treatment with 5 µM WA. Total lysates were immunoblotted for pRSK and total RSK expression. Actin was used as loading control. Bar graphs present relative density units of immunoblot signals. *p < 0.001, compared with control cells (**C**).

**Figure 5:**
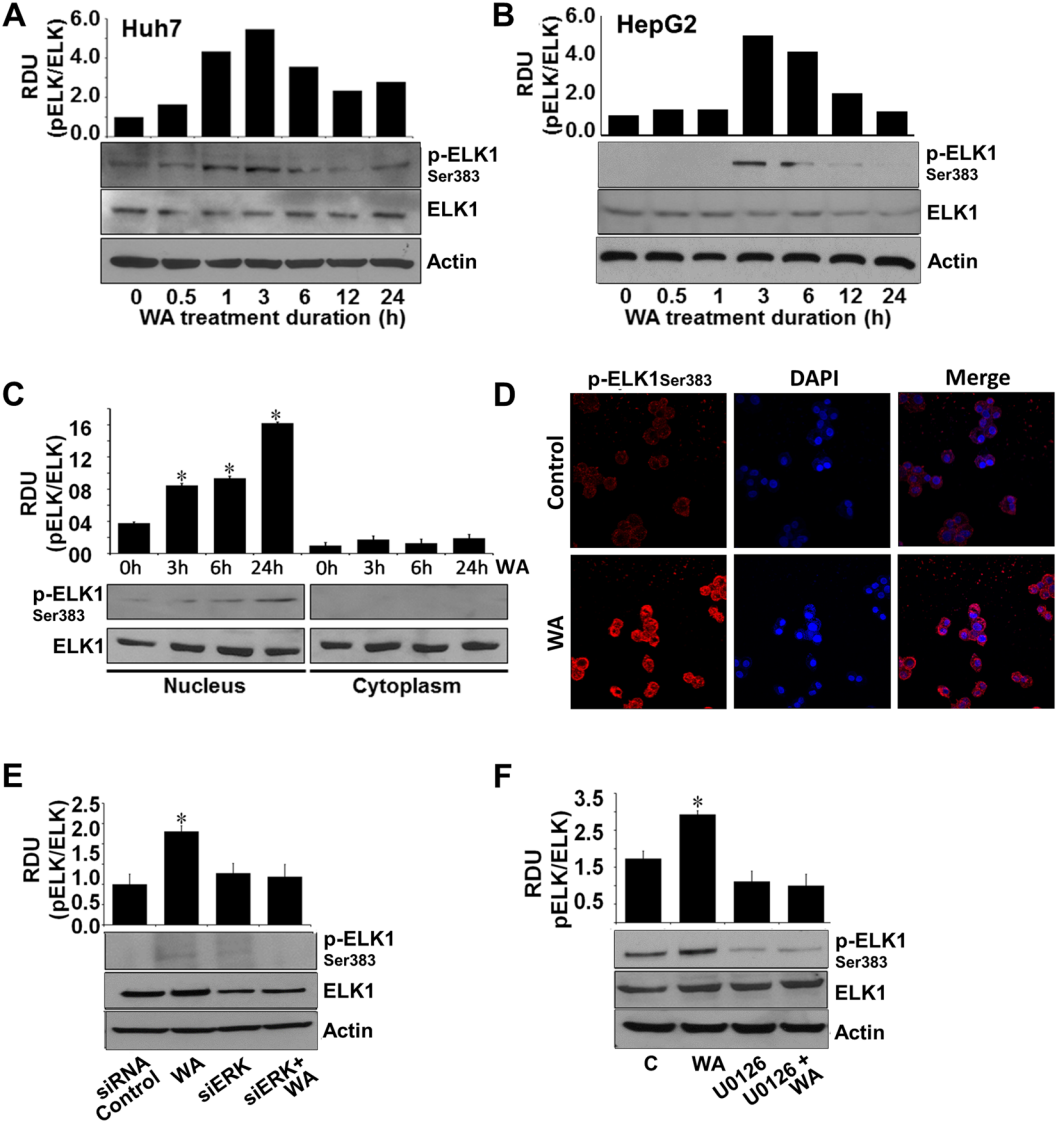
ERK is important for WA-mediated ELK1 activation in HCC cells. (**A**,**B**) Immunoblot analysis of phosphorylated-ELK1and total ELK1 in Huh7 and HepG2 cells treated with 5 µM WA as indicated. (**C**) Nuclear and cytoplasmic lysates from WA-treated HCC cells were immunoblotted for phosphorylated-ELK1and total ELK1. *p < 0.005, compared with 0 hour control cells. (**D**) HCC cells were treated with 5 µM WA and subjected to immunofluorescence analysis using anti-pELK1 antibodies. (**E**) HCC cells were transiently transfected with siERK-siRNA and treated with 5 µM WA as indicated and followed by immunoblot analysis of pELK1 and total ELK1 expression. *p < 0.001, compared with siRNA-control cells. (**F**) HCC cells were pretreated with 10 µM U0126 for 2 hours followed by treatment with 5 µM WA. Total lysates were immunoblotted for pELK1 and total ELK1 expression. Actin was used as loading control. Bar graphs present relative density units of immunoblot signals. *p < 0.01, compared with control cells (**C**).

Dysregulation of p90-ribosomal S6 kinase (RSK) in cancer cells controls multiple downstream signaling pathways and cellular processes acting as a potential tumor suppressor decreasing growth, invasion and migration of cancer cells^19–21^. Huh7 and HepG2 cells exhibited increased phosphorylation of RSK in response to WA treatment (Fig. 4A,B). RSK activation is controlled by canonical Ras/mitogen-activated protein kinase (MAPK) pathway therefore, we questioned whether ERK mediated RSK phosphorylation in WA-treated HCC cells. Towards this end, we silenced ERK (Fig. 4C) and investigated the impact of ERK silencing on WA-induced RSK activation in HCC cells. ERK silencing inhibited WA-induced RSK phosphorylation (Fig. 4E). Abrogation of ERK phosphorylation using ERK inhibitor, U0126 (Fig. 4D) also diminished RSK phosphorylation in WA-treated cells (Fig. 4F). Another important node of MAP kinase mediated anti-cancer cascade is the ETS domain transactivation factor Elk1 whose phosphorylation at serine 383 by MAP kinases triggers apoptotic induction and growth inhibition^22^. Transient increase in ELK-1 phosphorylation was observed in Huh7 and HepG2 cells treated with WA (Fig. 5A,B). Elevated expression of phosphorylated ELK1 was observed in nuclear fractions of WA-treated HCC cells in comparison to cytoplasmic fractions, whereas total ELK1 remained unaltered in nuclear and cytoplasmic fractions of WA-treated HCC cells (Fig. 5C). Immunofluorescence analysis of HCC cells showed nuclear localization of phosphorylated ELK1 upon WA treatment (Fig. 5D). Next, we investigated the connection of ERK in WA-mediated ELK1 activation using ERK siRNA and U0126 treatment. Pretreatment of HCC cells with ERK siRNA or U0126 inhibited WA-induced ELK1 phosphorylation showing a direct involvement of ERK (Figure E, F).

### Withaferin A induced upregulation of Death receptor 5 in HCC cells in an ERK-dependent manner

**Figure 6:**
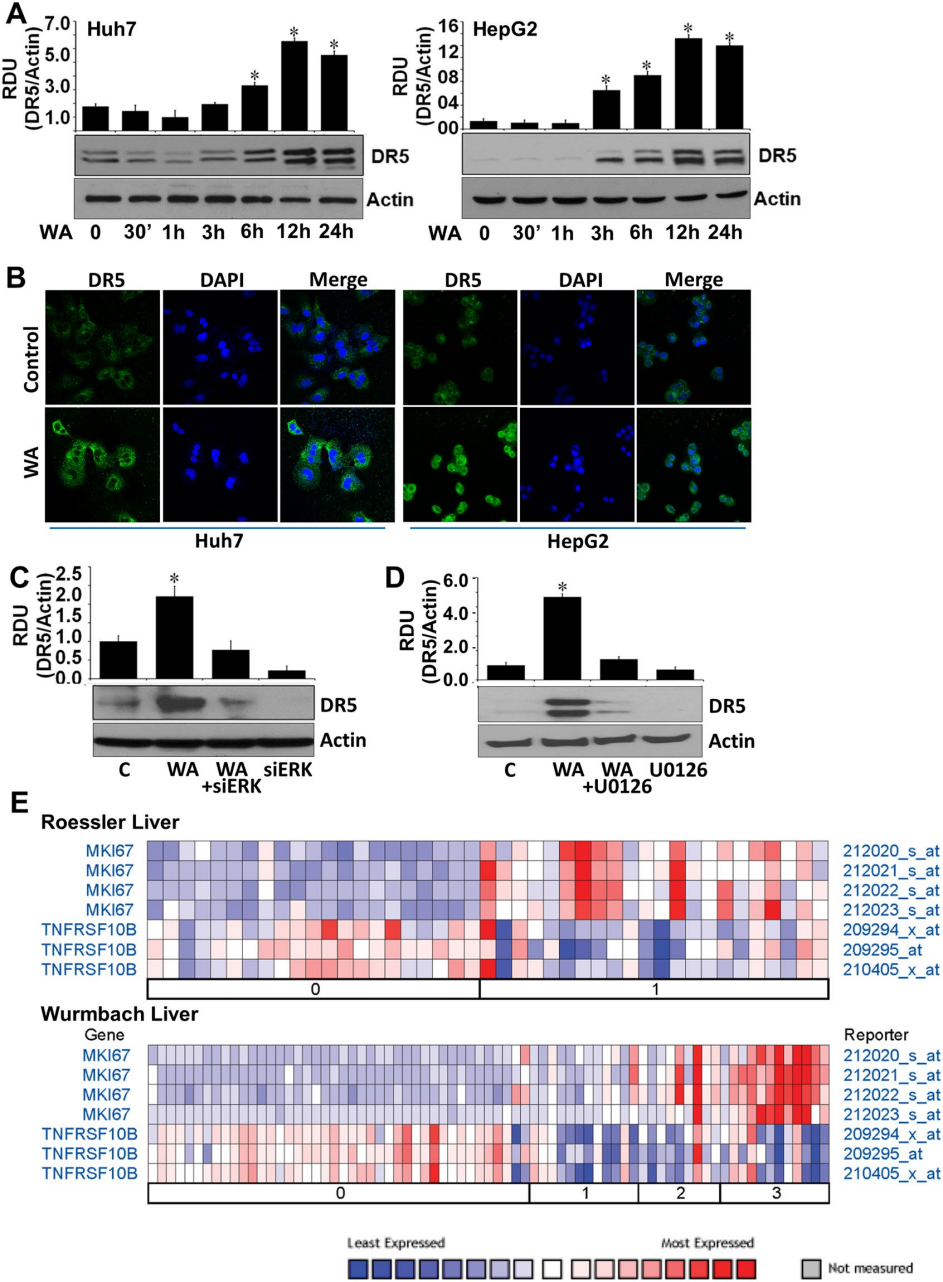
WA treatment increases DR5 expression in HCC cells in an ERK-dependent manner and DR5 and Ki67 exhibit inverse correlation. (**A**) Huh7 and HepG2 cells were treated with 5 µM WA and analyzed for DR5 expression in immunoblot analysis. *p < 0.01, compared with 0 hour control cells. (**B**) HCC cells were treated with 5 µM WA and subjected to immunofluorescence analysis using anti-DR5 antibodies. (**C**) HCC cells were transiently transfected with siERK-siRNA and treated with 5 µM WA as indicated and followed by immunoblot analysis of DR5 expression. *p < 0.005, compared with control cells (**C**). (**D**) HCC cells were pretreated with 10 µM U0126 for 2 hours followed by treatment with 5 µM WA. Total lysates were immunoblotted for DR5 expression. Actin was used as loading control. Bar graphs present relative density units of immunoblot signals. *p < 0.01, compared with control cells (**C**). (**E**) Roessler Liver and Wurmbach Liver datasets from the Oncomine were analyzed for DR5 and Ki67 expression.

Several bioactive and synthetic anti-cancer small molecules stimulate induction of apoptotic cascade via overexpression or activation of death receptor 5, a death domain-containing transmembrane receptor. Exploring the involvement of DR5 in WA function in HCC, we found that Huh7 and HepG2 cells exhibited a temporal increase in DR5 expression in response to WA treatment (Fig. 6A). Immunofluorescence staining of DR5 showed increased expression of DR5 in WA-treated cells in comparison to untreated cells (Fig. 6B). Involvement of ERK in WA-mediated DR5 upregulation was investigated using ERK siRNA and U0126 treatment. HCC cells pretreated with ERK siRNA or U0126 showed reduced levels of DR5 expression even in the presence of WA showing a direct involvement of ERK (Fig. 6C,D). Nuclear non-histone protein Ki-67 is widely used as a proliferation associated marker in cancer cells to guide adjuvant therapy decisions^23,24^. Ki-67 is an independent prognosis factor for HCC and increased expression of Ki-67 correlates with higher-grade malignancy and worse prognosis^25,26^. We analyzed existing microarray datasets to find whether there is any correlation between DR5 and Ki-67 in HCC using an online resource (Oncomine)^27^. We found that Wurmbach Liver dataset (75 samples, 19,574 measured genes, Human Genome U133 Plus 2.0 array) and Roessler Liver dataset (43 samples, 12,603 measured genes, Human Genome U133A 2.0 Array) showed an inverse correlation between DR5 and Ki67. High expression of Ki-67 was observed in HCC grade 1, 2 and 3 which correlated with low expression of DR5 while low Ki-67 expression correlated with high DR5 expression (Fig. 6E).

### Oral administration of Withaferin A inhibited HCC progression and modulated activation of ERK1/2-ELK1-RSK-DR5 axis *in vivo*

**Figure 7:**
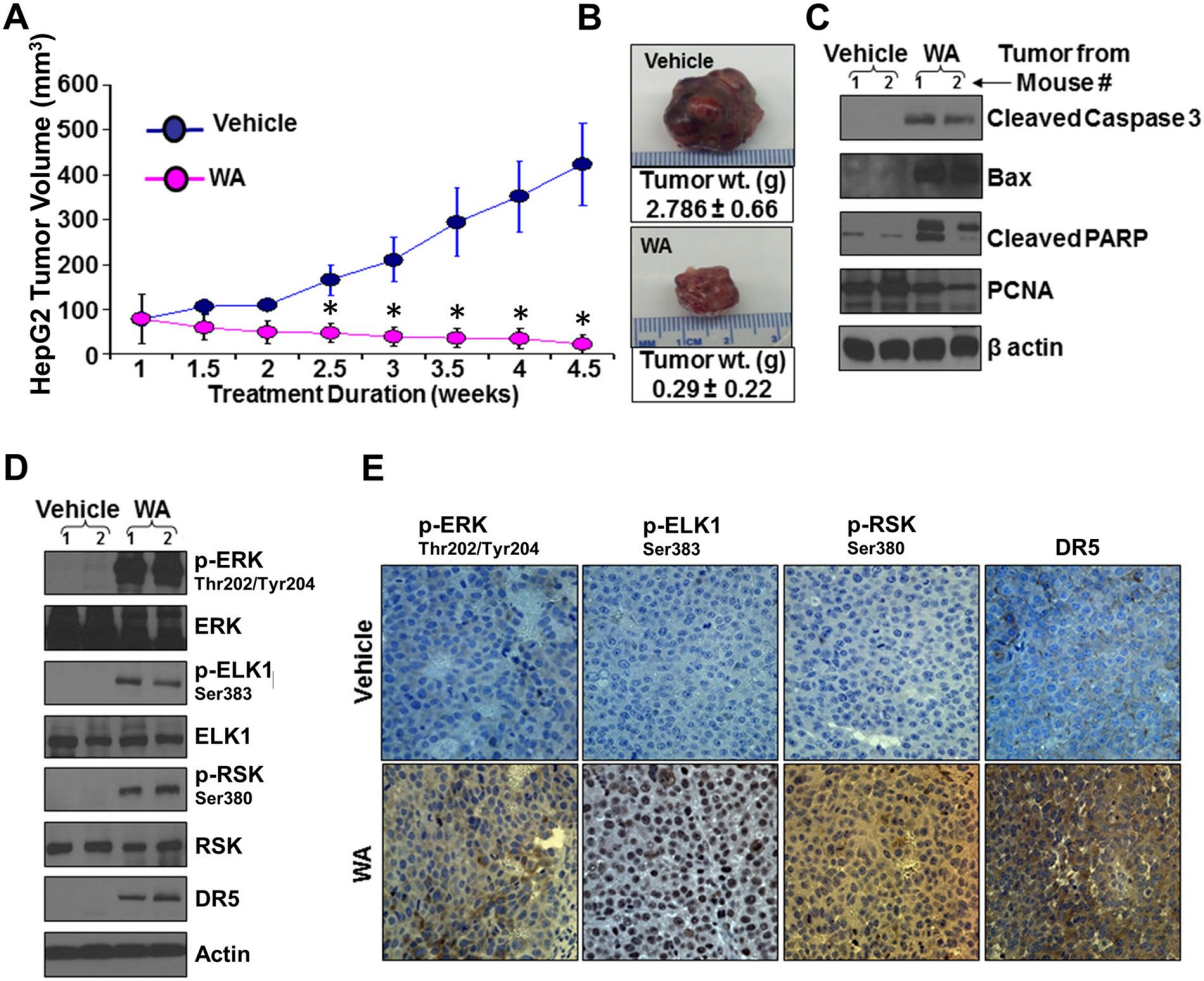
Withaferin A treatment inhibits HCC growth in nude mice. HepG2 cells-derived tumors were developed in nude mice and treated with vehicle or WA. (**A**) Tumor growth was monitored by measuring the tumor volume for 4.5 weeks (n = 8 mice per group). *p < 0.001, compared with vehicle-treated group. (**B**) Tumors weight and volume were measured at the end of treatment period. Average tumor weight and representative tumor images are shown here. (**C**) Tumor lysates were immunoblotted for the expression of cleaved caspase 3, Bax, cleaved PARP and PCNA as indicated. (**D**) Tumor lysates were immunoblotted for the expression of DR5, phosphorylated and total ERK, ELK1, RSK as indicated. β-Actin antibody was used as loading control. (**E**) Tumor samples were subjected to immunohistochemical analysis using phospho-ERK1, phospho-ELK1, phospho-RSK and DR5 antibody.

We aspired to investigate the *in vivo* significance of our *in vitro* findings by evaluating the effect of WA on HCC progression in athymic nude mice models. WA treatment significantly inhibited growth of HepG2-derived tumors in comparison to the vehicle-treated group (Fig. 7A,B). WA-treated HepG2 tumors showed higher levels of cleaved caspase 3, Bax, cleaved PARP and lower level of PCNA (Fig. 7C). Corroborating the *in vitro* findings showing the involvement of ERK-RSK-ELK1-DR5 axis in WA-function, tumors from WA-treated mice exhibited higher phosphorylation levels of ERK, RSK, ELK1 in comparison to tumors from vehicle-treated mice. Elevated DR5 expression was also observed in WA-treated tumors (Fig. 7D). Immunohistochemical analyses also showed higher expression of DR5 and elevated levels of phosphorylated-ERK, -ELK1 and -RSK in tumors from WA-treated group in contrast to the vehicle-treated group (Fig. 7E).

### Withaferin A prevented tumor growth and tumor burden in diethylnitrosamine (DEN)-induced hepatocarcinogenesis model

**Figure 8:**
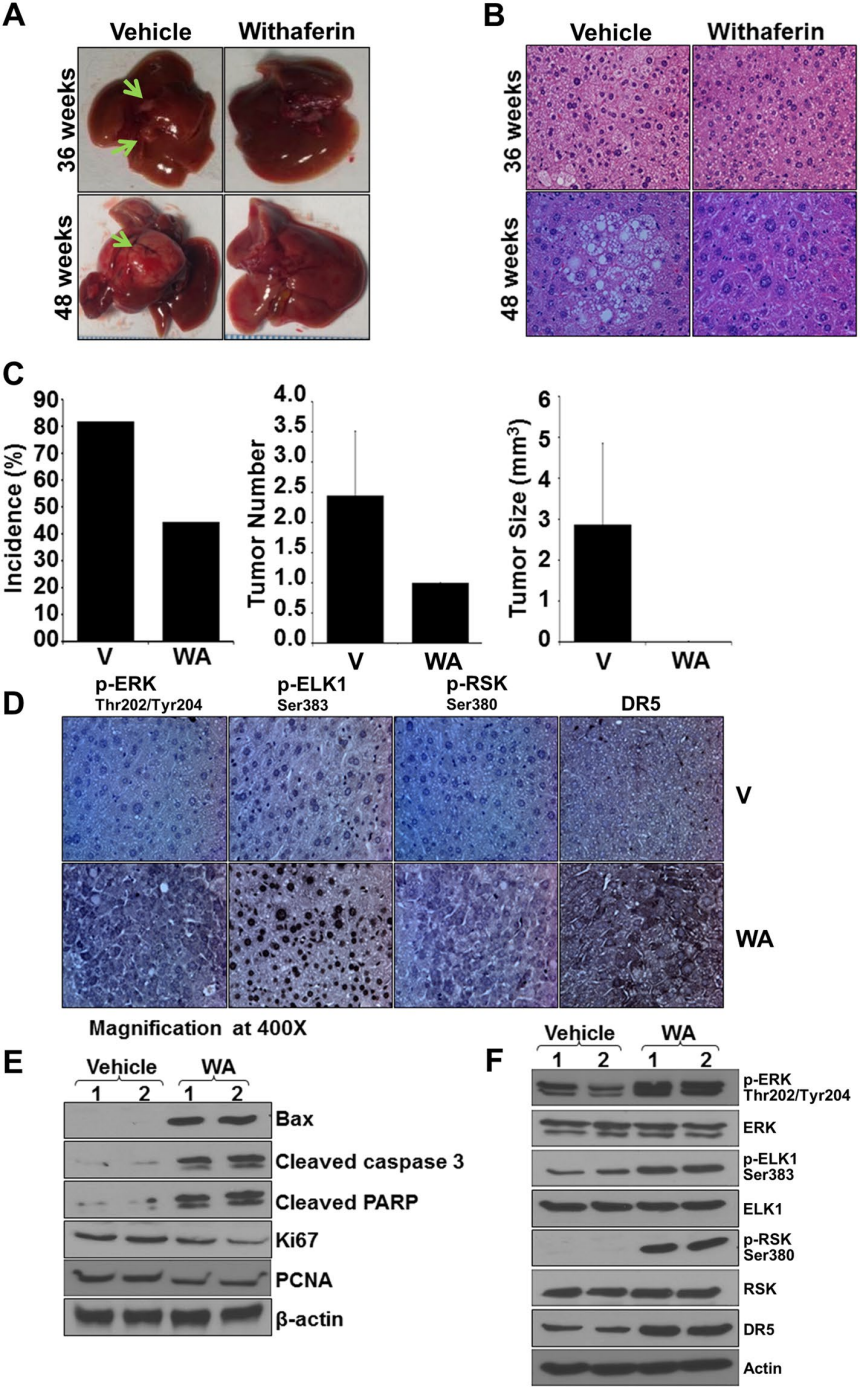
Withaferin A treatment inhibits DEN-induced liver tumor formation in C57BL/6 mice. C57BL/6 mice were given intraperitoneal injection of DEN (25 mg/kg/body weight) to develop liver tumors. Tumor bearing mice were randomized to vehicle or WA treatment groups and oral administration of vehicle or WA continued for 36 or 48 hours. (**A**) Representative pictures showing macroscopic changes in the liver from DEN-C57BL6 mice treated with vehicle or WA for 36 and 48 weeks. Arrows point to tumors in untreated group. (**B**) Representative images of H&E staining of liver tumors from vehicle and WA treated groups. (**C**) Bar diagram for (i) tumor incidence, (ii) tumor multiplicity and (iii) tumor size for vehicle-treated and WA-treated DEN-C57BL6 mice (n = 8 mice/group). (**D**) Tumors were subjected to immunohistochemical analysis using pERK, pELK1, pRSK, and DR5 antibodies. Columns, mean (n = 8). (**E**) Tumor lysates were immunoblotted for Bax, cleaved Caspase 3, cleaved PARP, PCNA and Ki67 as indicated. (**F**) Tumor lysates were subjected to immunoblot analyses using phosphorylated and total ERK, ELK1, RSK and DR5 as indicated. β actin was used as loading control.

Enthused with the *in vivo* efficacy of WA in HepG2-xenograft models, we investigated whether WA administration could prevent liver cancer in a carcinogen-induced HCC model. Towards this end, 14-day old C57BL/6 mice were injected with DEN followed by treatment with vehicle or WA and tumor incidence and tumor growth was assessed after 36 and 48 weeks. Interestingly, WA treatment prevented DEN-induced liver tumor formation in C57BL/6 mice and significantly reduced the tumor size as compared to untreated group as early as 36 weeks-post treatment (Fig. 8A,B). Significant decrease in tumor incidence, tumor number (multiplicity) and tumor size was observed in WA-treated mice in comparison to vehicle-treated mice (Fig. 8C). Immunohistochemical analyses showed that tumors from WA-treated mice exhibited higher number of tumor cells showing increased expression of phosphorylated-ERK, -RSK, -ELK1, and DR5 as compared to tumors from untreated group (Fig. 8D) providing physiologic relevance to our *in vitro* findings. Further, we investigated the key molecules of WA-functional axis utilizing tumor tissues from DEN-induced HCC model. WA-treated HCC tumors showed upregulation of apoptosis signaling molecules Bax, Cleaved caspase 3, Cleaved PARP and downregulation of cell proliferation signaling molecules such as PCNA and Ki67 (Fig. 8E). Also, liver tumor tissues from WA-treated mice showed increased phosphorylation of ERK, RSK, ELK1 as well as upregulation of DR5 expression compared with untreated group (Fig. 8F). Collectively, these results present *in vitro* as well as *in vivo* evidence supporting the anti-HCC efficacy of WA and show the involvement of ERK as an important mediator for WA-induced RSK/ELK1 activation and DR5 upregulation.

## Discussion

Incidence of HCC has more than tripled since 1980 and is associated with more than 600,000 cancer-related deaths worldwide each year^4^. Many drugs, small molecules, cytotoxic as well as targeted agents have been examined to inhibit HCC growth and progression in recent years. Conventional cytoxic chemotherapy regimens showed concerns related to toxicity and lack of survival benefits^28^. Amongst the targeted therapies tested for HCC, sorafenib, an oral multikinase inhibitor, prolonged the median overall survival for three months longer than placebo^5,6^ and currently, is the only approved agent for the treatment of advanced HCC. Several molecular targeted agents have been tested clinically since the approval of sorafenib but none of them achieved desirable outcome. With the incessant rise in HCC incidence, high HCC-associated mortality and lack of effective therapies, it is necessary to develop new effective therapeutic strategies for HCC. We recognize that an ideal therapeutic strategy to target HCC should be safe, highly efficacious, and non-toxic therefore, we focused on a bioactive small-molecule agent withaferin A (WA) to effectively inhibit HCC. We found that WA treatment effectively inhibits growth as well as migration and invasion potential of HCC cells whereas normal hepatocytes remained unaffected (data not shown). Elucidating the molecular signaling network mediating WA action is fundamental to establish surrogate biomarkers, a task essential for the clinical development of this bioactive molecule. We discovered an interesting pathway where WA-induced ERK/RSK led to a concomitant activation of ELK1 and DR5 in HCC cells. Our results also show that oral administration of WA inhibits HepG2-xenografts in athymic nude mice and DEN-induced-HCC in C57BL/6 mice and these *in vivo* studies provide further evidence of the integral involvement of ERK/RSK, Elk1 and DR5.

We report an interesting role of ERK in HCC as WA, a bioactive compound that effectively inhibits HCC also increases the phosphorylation of ERK. Although ERK is generally considered a survival-signaling pathway, its role in apoptosis induction has also been noted^29,30^ depending on the kinetics and duration of ERK activation. Transient ERK activation impedes cell death^31^ whereas sustained ERK activation results in apoptotic cell death^29^ and we noted that WA causes a prolonged ERK activation in HCC cells. We also show that WA activates RSK in an ERK-dependent manner which has been implicated in inhibition of apoptosis attained via phosphorylation of Bad, CEBPβ and DAPK^20^, as well as induction of apoptosis by phosphorylating Nur77^32^. ERK can mediate direct phosphorylation of Elk1^33^ which is recently reported as a transcription regulator of DR5^34^. We found that WA activates ELK-1 and DR5 in an ERK-dependent manner. We also observed an inverse correlation between DR5 and Ki67 where grade 0 HCC exhibit high DR5/low Ki67 and high-grade HCC exhibit low DR5/high Ki67 indicating that strategies to increase DR5 expression using Pro-Apoptotic Receptor Agonists (PARAs) might be useful in targeting HCC. Although activating death receptors to attain tumor growth inhibition has been investigated, not much progress has been achieved owing to the toxic effects of death receptor ligands-FASL and TNF. In addition, many PARAs [recombinant human TRAIL (dulanermin, Amgen/Genentech), lexatumumab (Human Genome Sciences), conatumumab (Amgen), drozitumab (Genentech), tigatuzumab (Daiichi-Sankyo) and LBY135 (Novartis)], alone and in combination, have been tested in phase 1 and 2 clinical trials^35,36^. While the phase 1 and 2 trials with these PARAs have been encouraging, no full phase 3 studies have been performed suggesting an imminent need for novel PARAs with improved properties. Our studies show that WA can act as an effective, bioactive PARA. In conclusion, our study deciphered a novel mechanism whereby WA achieves effective HCC inhibition and provides correlative biomarkers, which can prove useful in further development of WA as a therapeutic regimen for HCC.

## Materials and Methods

### Cell culture and reagents

HepG2 cells (ATCC, Manassas, VA) derived from a human hepatoblastoma^37^ and Huh7 cells derived from a well-differentiated HCC^38^ were cultured in minimum essential medium (ATCC) and Dulbecco’s modified Eagle’s medium (Invitrogen, Carlsbad, CA), respectively. Withaferin A (WA) was purchased from Calbiochem EMD Millipore (Billerica, MA). Antibodies for phospho-ERK, phospho-ELK1, phospho-RSK, phospho-p38, ERK, ELK1, RSK, DR5, Cleaved Caspase 3, Bax, cleaved-PARP, total-PARP, PCNA and β-actin were purchased from Cell-Signaling Technology (Danvers, MA). U0126 was procured from Sigma-Aldrich (St. Louis, MO).

### Apoptosis, cell-viability, clonogenicity and anchorage-independent growth assay

HCC cells were treated with WA and apoptosis was quantified by Hoechst staining^39^. Cell viability assay was performed^40^ by estimating reduction of XTT (2, 3-bis(2-methoxy-4-nitro-5-sulfophenyl)−2H-tetrazolium-5-carboxyanilide), using a commercially available kit (Roche Applied Science, Indianapolis, IN). Clonogenicity assay was performed according to our published protocol^41^. Anchorage-independent growth assay was performed according to our published protocol^40,42^.

### Spheroid migration, ECIS-migration and invasion assay

Spheroid migration was performed according to our published protocol^43^. HCC spheroids were treated with WA and migration of cells from spheroids was observed under a light microscope. Invasion assay was performed^44,45^ using matrigel invasion chambers (BD Biocoat Cellware, San Jose, CA). Migration and invasion of HCC cells in presence of WA were also measured in real-time by using Electric cell-substrate impedance sensing (ECIS) (Applied BioPhysics, Troy, NY) technology following our previously established protocol^46^.

### Phospho-Antibody Array Analysis

HCC cells were treated with WA and the phospho-antibody array analysis was performed using the Proteome Profiler Human Phospho-Kinase Array Kit ARY003 from R&D Systems (Minneapolis, MA) according to the manufacturer’s instructions. Array images were analyzed using the GeneTools image analysis software (Syngene).

### Transfection, siRNA, sub-cellular fractionation and immunoblotting

HCC cells were transfected with 100 nM of validated siRNA (control siRNA or ERK1/2 siRNA) (Santa Cruz Biotecnology, Dallas, TX) followed by WA treatment. Whole cell lysates, cytosolic and nuclear fractions were prepared following our previously published protocol^43^ and subjected to immunoblot analysis using specific antibodies. Immunodetection was performed using enhanced chemiluminescence (ECL system, Amersham Pharmacia Biotech Inc., Arlington Heights, IL).

### Immunofluorescence and confocal imaging

HCC cells (2 × 10^3^ cells/well) were plated in 4-well chamber slides (Nunc, Rochester, NY) followed by treatment with WA and subjected to immunocytochemistry using our published protocol^47^. Fixed and immunofluorescently stained cells were imaged using a Zeiss LSM510 Meta laser scanning confocal system configured to a Zeiss Axioplan 2 upright microscope with a 63XO (NA 1.4) plan-apochromat objective (Zeiss, Dublin, California, USA).

### HepG2-cell-xenografts in nude mice

HepG2 (5 × 10^6^) cells in 0.1 ml of HBSS were injected subcutaneously into the right gluteal region of 4–6-week-old male athymic nude mice, (Harlan Laboratories, Indianaplois, IN). Two weeks after initial implantation, mice were randomized to vehicle and treatment arm (8 mice per group). Mice were gavaged with vehicle (10% DMSO, 40% cremophor-EL and 50% PBS) or vehicle containing 4 mg/kg body weight withaferin A (ChromaDex Inc., Irvine, CA) daily for 5 weeks. The dose and route of WA administration was selected from previous studies documenting *in vivo* efficacy of WA^48^. Tumors were measured regularly^43,49^ and all the animals were sacrificed after 5 weeks of treatment. Tumors were dissected, weighed, fixed in 10% neutral-buffered formalin and subjected to further analyses by immunoblot, RT-PCR and immunohistochemistry. All animal studies were approved by the Institutional Animal Care and Use Committee, University of Maryland School of Medicine, Office of Welfare Assurance, Baltimore, Maryland.

### DEN-induced hepatocarcinogenesis in C57BL/6 mice

Two weeks old male C57BL/6 mice (Harlan Laboratories, Indianapolis, IN) were injected with Diethylnitrosamine (DEN) (25 mg/kg body weight) via intraperitoneal (i.p.) route and mice were grouped randomly into two experimental groups (10 mice per group)-Vehicle and WA. Mice were gavaged with vehicle (10% DMSO, 40% cremophor-EL and 50% PBS) or vehicle containing 4 mg/kg body weight WA (ChromaDex Inc., Irvine, CA) daily for 48 weeks. At the end of experiment, tumors were dissected, counted for tumor multiplicity, measured for volume and weight. All animals were sacrificed after 48 weeks of treatment. At least five random, non-overlapping representative images from each tumor section from tumors of each treatment group were captured using ImagePro software for quantification of pERK, pElk1, pRSK and DR5 expression. All animal studies were approved by the Institutional Animal Care and Use Committee, University of Maryland School of Medicine.

### Statistical Analysis

All experiments were performed thrice in triplicates. Statistical analysis was performed using Microsoft Excel software. Significant differences were analyzed using student’s *t* test and two-tailed distribution. Data were considered to be statistically significant if *p* < 0.05. Data were expressed as mean ± SE between triplicate experiments performed thrice.

All methods were performed in accordance with the relevant guidelines and regulations of Johns Hopkins University.
